# Supplementary material for: Whole-genome comparisons identify repeated regulatory changes underlying convergent appendage evolution in diverse fish lineages
Source: bioRxiv. 2023 Jan 31:2023.01.30.526059. Preprint. [Version 1] doi: 10.1101/2023.01.30.526059 (PMC9915506; doi:10.1101/2023.01.30.526059)
Supplement: Supplement 5 [file media-5.pdf]

Supplemental Figures & Text

Figure 1–figure supplement 1

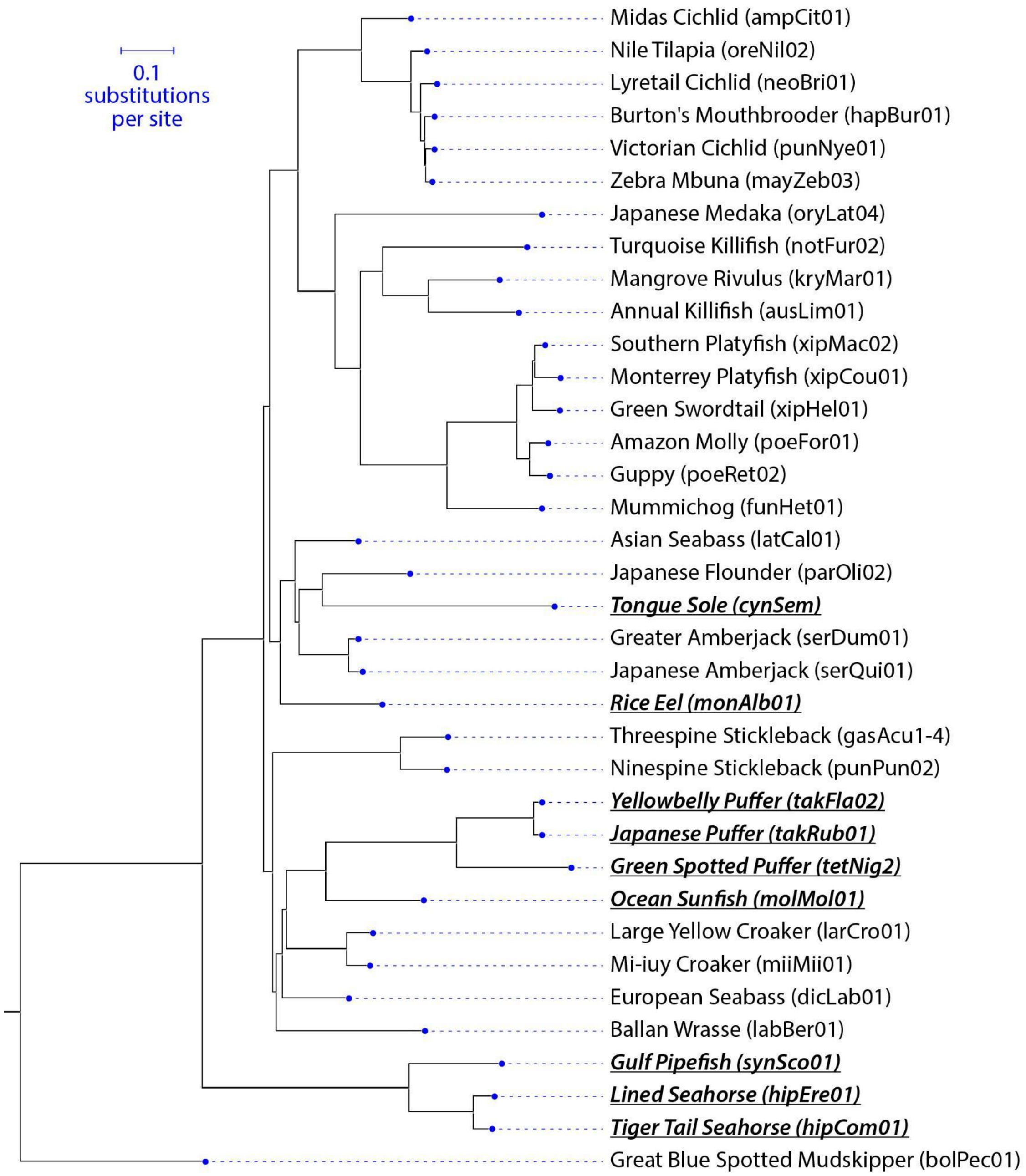

Figure 1–figure supplement 1. Branch length-calibrated tree. Common names (with associated genome assembly abbreviations) for all 36 species in the computational screen are listed (also see Supplemental File 1). Target species are underlined and italicized. The topology of this tree is based on a consensus of several recent studies (Alfaro *et al.*, 2018; Hughes *et al.*, 2018; Mu *et al.*, 2022).

Figure 1–text supplement 1

Figure 1–text supplement 1. Branch lengths for trees shown in Fig. 1A and Fig. 1–fig sup 1

```
ALPHABET: A C G T
ORDER: 0
SUBST_MOD: REV
BACKGROUND: 0.190773 0.360458 0.235183 0.213586
RATE_MAT:
  -1.168358    0.296539    0.622435    0.249384
   0.156944   -0.785009    0.199225    0.428841
   0.504898    0.305345   -1.025896    0.215653
   0.222747    0.723732    0.237459   -1.183938
TREE:
((((((ampCit01:0.0887886,(oreNil02:0.0232145,(neoBri01:0.0255143,(hapBur01:0.0112809,(punNye01:0.0105135,mayZe
b03:0.00683988):0.00242368):0.00857014):0.0188007):0.0940882):0.119616,(oryLat04:0.385762,((notFur02:0.267095,
(ausLim01:0.166114,kryMar01:0.130003):0.0857237):0.042407,((((xipMac02:0.0143759,xipCou01:0.0434946):0.0039567
9,xipHel01:0.0468436):0.0309337,(poeFor01:0.0306962,poeRet02:0.0337162):0.0245541):0.131882,funHet01:0.174194)
:0.164058):0.0487246):0.0707918):0.0535661,((latCal01:0.114761,((parOli02:0.160689,cynSem:0.433672):0.0437825,
(serQui01:0.0202901,serDum01:0.0129067):0.0946104):0.00742124):0.0278147,monAlb01:0.188107):0.0202366):0.01178
74,((gasAcu14:0.0832039,punPun02:0.0819135):0.241185,((((((takFla02:0.00916032,takRub01:0.0091555):0.146708,te
tNig2:0.212735):0.248094,molMol01:0.180985):0.0734705,(larCro01:0.0435642,miiMii01:0.0373942):0.114976):0.0082
0025,dicLab01:0.119023):0.0125103,labBer01:0.276969):0.00632666):0.0188741):0.116492,(synSco01:0.170947,(hipEr
e01:0.0331536,hipCom01:0.0298633):0.12246):0.392849):0.343345,bolPec01:0.343345);
```

Figure 1–figure supplement 2

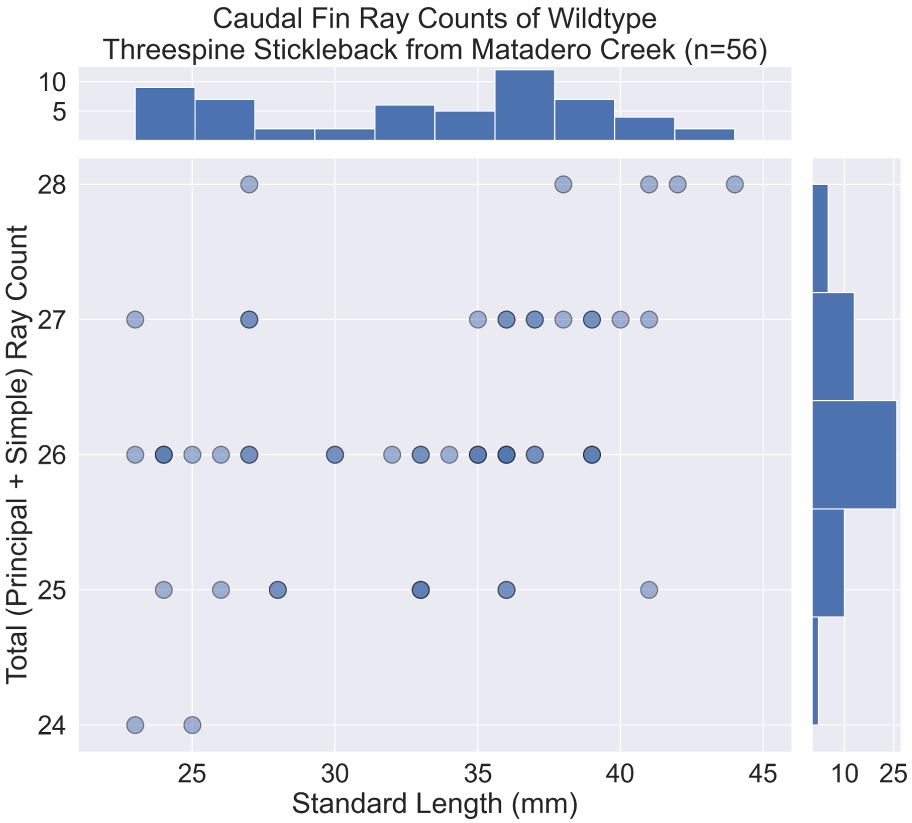

Figure 1–figure supplement 2. Caudal ray counts in wildtype Matadero Creek Threespine Stickleback. Total *segmented principal* and *unsegmented simple* caudal ray (lepidotrichia) counts plotted against fish standard length. Data are from n=56 lab-reared wildtype Threespine Stickleback descendants of fish collected from Matadero Creek, California.

Figure 4–figure supplement 1

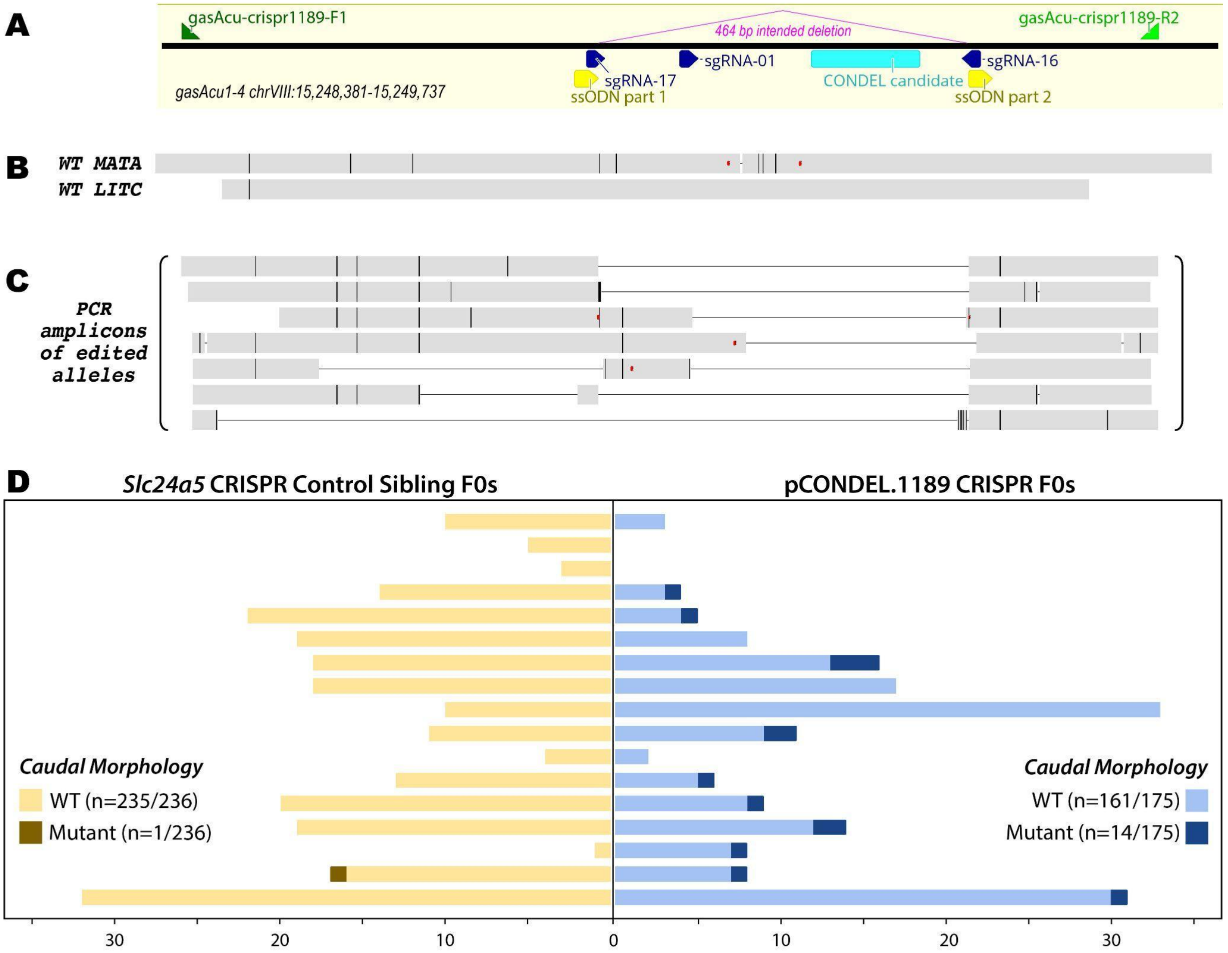

Figure 4–figure supplement 1. CRISPR-Cas9 deletions and morphological effects of recapitulating the pCONDEL.1189 evolutionary lesion. A, Schematic of the CRISPR-targeted pCONDEL.1189 genomic region in Threespine Stickleback (`gasAcu1-4`) genomic space. PCR primers are shown as green triangles, sgRNAs are shown as dark blue triangles, and the injected 60 bp single-stranded DNA oligonucleotide is a concatemer of the two yellow triangles (Table S5). **B**, Sanger-sequenced amplicons from wildtype (WT) descendants of Matadero Creek, California (MATA) and Little Campbell River, Canada (LITC). **C**, Examples of Sanger-sequenced amplicons recovered from crispants injected with Cas9 protein and the reagents illustrated above (see Table S3 for injected concentrations and Table S5 for oligonucleotide sequences). In **B** and **C**, black vertical lines indicate nucleotide substitutions relative to the `gasAcu1-4` reference sequence; horizontal black lines represent deleted sequence; red dots denote insertions; and light gray bars illustrate reference-identical sequence. **D**, Paired bar graphs showing the number of scored *Slc24a5* CRISPR control siblings on the left, and pCONDEL.1189 CRISPR F0s on the right for 17 clutches (each row represents one clutch). Darker and lighter shaded portions of the bar graphs, respectively, indicate the number of individuals with ectopic dorsal segmented rays (as in Fig. 4C) or with wildtype caudal morphology (as in Fig. 4B).

Figure 4–figure supplement 2

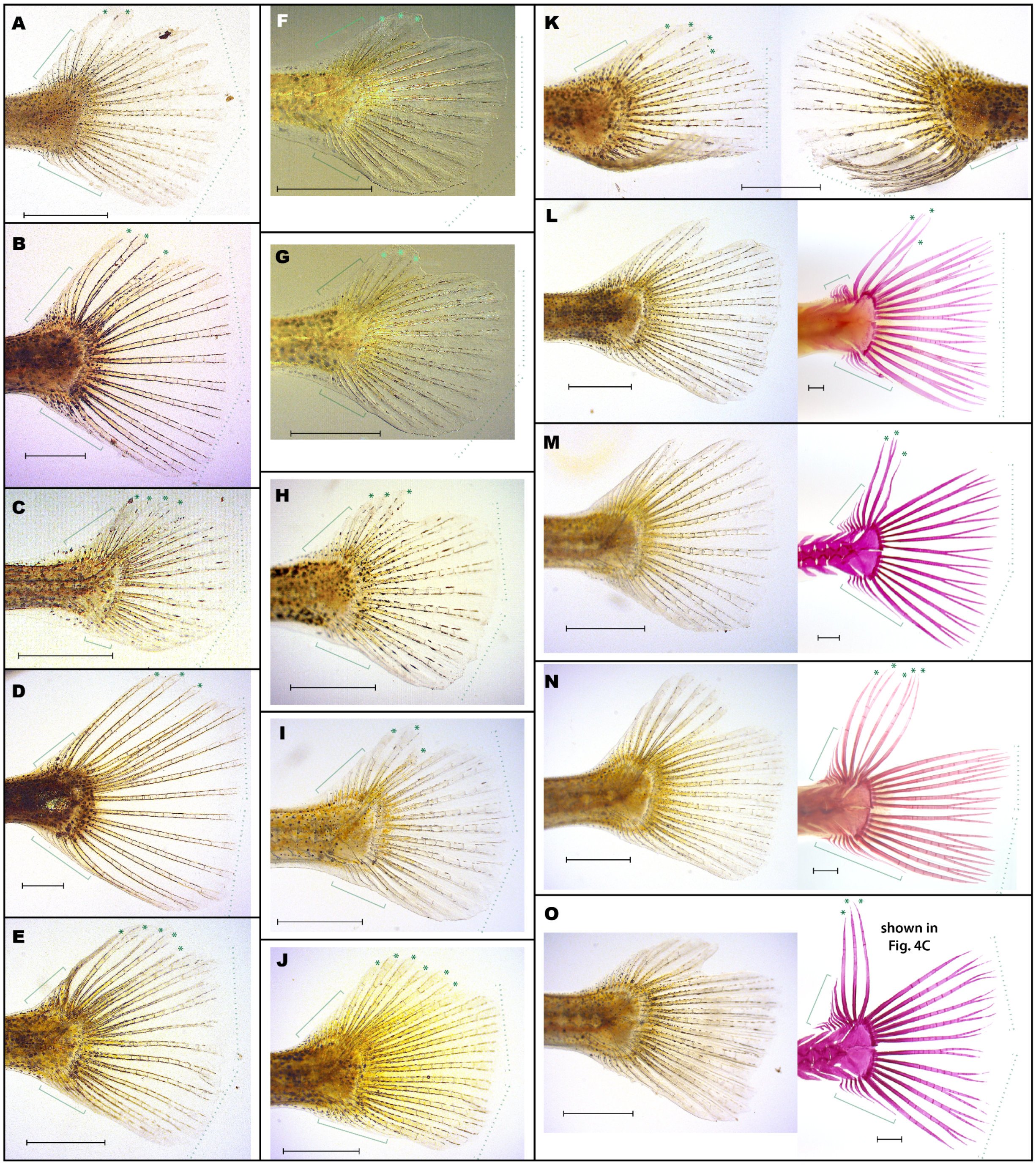

Figure 4–figure supplement 2. Fish from pCONDEL.1189-targeting experiments with ectopic dorsal rays. A, The single *Slc24a5*-targeted control sibling (out of 236 total) that developed ectopic dorsal caudal rays. **B-O**, All 14 enhancer deletion F0 crispant fish (out of 175 total) reported in Fig. 4–fig sup 1D to have ectopic dorsal rays. Panel **K** shows the left and right sides of one fish with a curled tail fin. **L-O** each shows two photos of a single fish – at an earlier and later developmental stage from left to right, respectively. Dashed brackets denote typical, segmented *principal* rays on the dorsal and ventral halves of the tail fin; solid brackets denote unsegmented *simple* rays; asterisks (*) denote ectopic segmented rays. All scale bars are 1 mm.

Figure 4–figure supplement 3

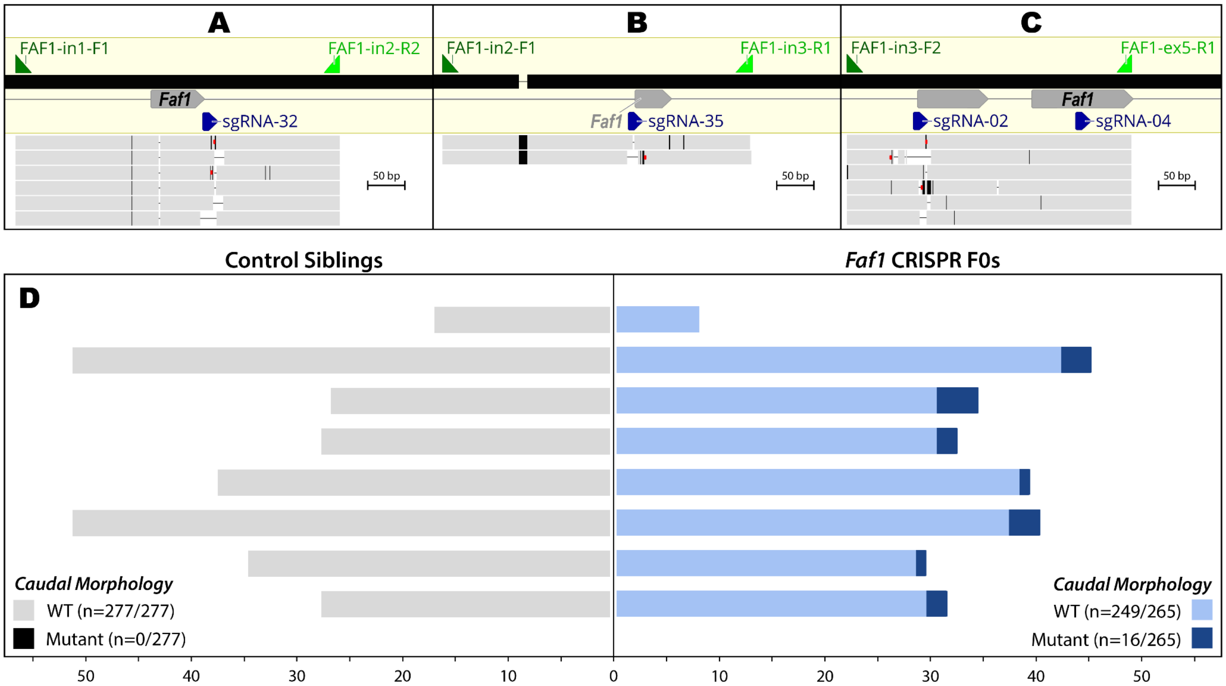

Figure 4–figure supplement 3. CRISPR-Cas9 lesions and morphological effects of disrupting *Faf1* coding regions. A-C, Schematics of exons 2-5 of *Faf1* in Threespine Stickleback (`gasAcu1-4`) sequence space. PCR primers are shown as green triangles. Examples of PCR amplicons from fish injected with Cas9 protein and all 4 of the sgRNAs (dark blue triangles) illustrated (see Table S3 for injected concentrations and Table S5 for oligonucleotide sequences). Black vertical lines indicate nucleotide substitutions relative to the `gasAcu1-4` reference sequence; horizontal black lines represent deleted/missing sequence; red dots denote insertions; and light gray bars illustrate reference-identical sequence. Numerous editing events were detected at the target of sgRNA-32 in **A** and sgRNA-02 in **C**. Very few editing events were detected at the target of sgRNA-35 in **B**, and no conclusive editing events were detected at the target of sgRNA-04 in **C**. **D**, Paired bar graphs showing the number of scored control siblings on the left, and *Faf1*-targeted siblings on the right, from 10 clutches. Two sets of smaller clutches were combined for practical husbandry purposes, so 8 total rows are shown. Darker and lighter shaded portions of the bar graphs, respectively, indicate the number of individuals with ectopic dorsal segmented rays (as in Fig. 4F) or with wildtype caudal morphology (as in Fig. 4E).

Figure 4–figure supplement 4

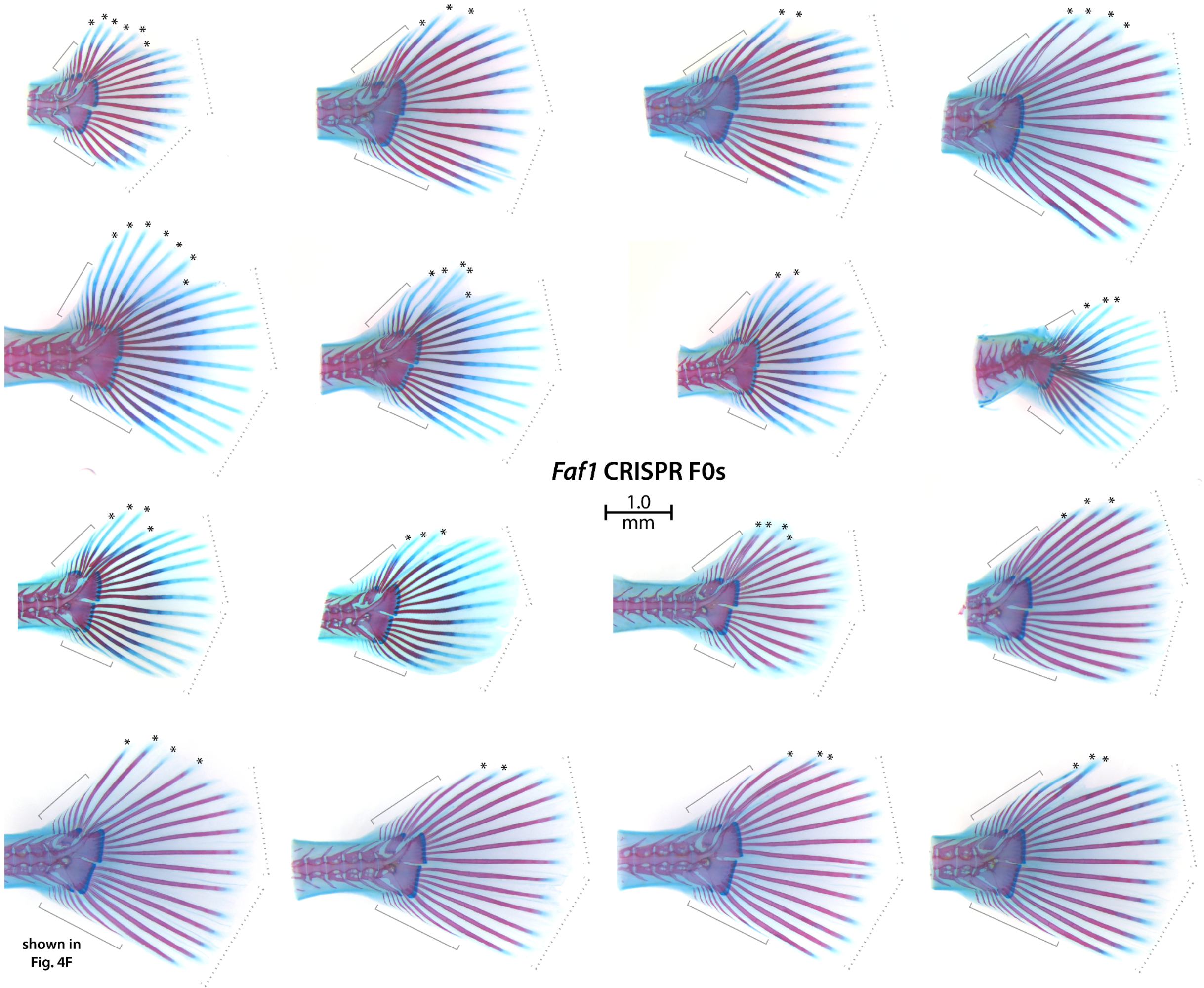

Figure 4–figure supplement 4. Fish from *Faf1*-targeting experiments with ectopic dorsal rays. All 16 *Faf1*-targeted F0 crispant fish reported in Fig. 4–fig sup 3D to have ectopic dorsal rays. Dashed brackets denote typical, segmented *principal* rays on the dorsal and ventral halves of the tail fin; solid brackets denote unsegmented *simple* rays; asterisks (*) denote ectopic segmented rays.

Figure 4–figure supplement 5

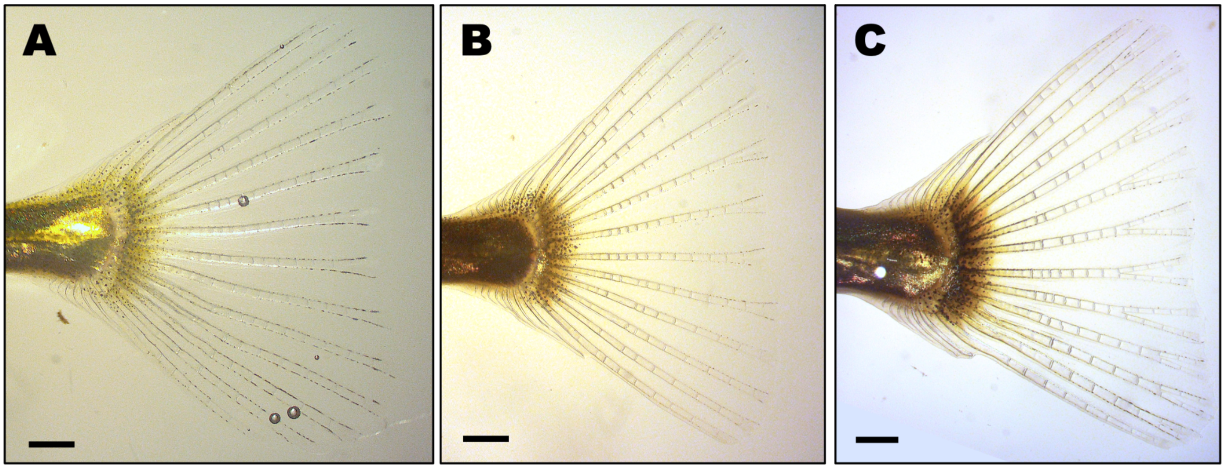

Figure 4–figure supplement 5. Wildtype caudal morphology in fish with stably transmitted pCONDEL.1189 deletion alleles. Mosaic F0 CRISPR founders were intercrossed to generate F1 fish carrying different combinations of wildtype and pCONDEL.1189 deletion alleles. Representative fish from three clutches that all had wildtype caudal morphology, including individuals with two intact pCONDEL.1189 alleles (**A**), fish homozygous for a 927 bp pCONDEL.1189 deletion allele (**B**), and compound heterozygotes carrying 927bp and 229bp pCONDEL.1189 deletion alleles (**C**). All deletion alleles in the fish shown in **B** and **C** remove the entire 135 bp pCONDEL.1189 sequence. All scale bars are 1 mm.

Supplemental Tables

Table S1: References for pelvic and caudal fin status of key target and outgroup species.

Scientific name	Common name	# of pelvic fins	Total # of caudal fin rays	Reference
Cynoglossus crepida	Congener of Tongue Sole	1	8	[1]
Cynoglossus semilaevis	Tongue Sole	1	10	[2]
Cynoglossus sp.	Congener of Tongue Sole	1	8-12	[3]
Cynoglossus westraliensis	Congener of Tongue Sole	1	8	[4]
Cynoglossus yokomaru	Congener of Tongue Sole	1	10	[5]
Dicentrarchus labrax	European Seabass	2	16-18 principal + numerous simple rays	[6]
Dicentrarchus labrax	European Seabass	[not mentioned]	17 principal, 29-31 simple	[7, 8]
Gasterosteus aculeatus	Threespine Stickleback (Matadero Creek)	2	$\geq$24	This study: see Fig 1–Sup. Fig.2
Gasterosteus aculeatus	Threespine Stickleback	[not mentioned]	22-31 for fish $\geq$15mm	[9]
Gasterosteus aculeatus	Threespine Stickleback	[not mentioned]	24	[10]
Hippocampus comes	Tiger Tail Seahorse	0	[not mentioned]	[11]
Hippocampus erectus	Lined Seahorse	0	[not mentioned]	[11]
Hippocampus sp.	Congener of seahorses	0	0-3	[12]
Larimichthys crocea	Yellow Croaker	2	35	[13]
Mola mola	Ocean Sunfish	0	0	[11, 14, 15]
Monopterus albus	Rice Eel	0	0	[16, 17]
Oryzias latipes	Japanese Medaka	[not mentioned]	20-24	[18]
Paralichthys olivaceus	Japanese Flounder	2	18-19	[19]
Paralichthys olivaceus	Japanese Flounder	2	[not mentioned]	[20]
Paralichthys sp.	Congener of Japanese Flounder	2	18	[21]
Seriola dumerili	Greater Amberjack	2	39	[22]
Syngnathus scovelli	Gulf Pipefish	0	[not mentioned]	[11]
Syngnathus schlegeli	Congener of Gulf Pipefish	[not mentioned]	9-10	[23]
Tetraodontoidea	Superfamily of pufferfish	0	9-11	[14]
Tetraodontidae	Family of pufferfish	0	10	[11]
Tetraodontidae	Family of pufferfish	[not mentioned]	11	[8]

Table S2: Tissue, cell line, and genomic DNA sources. GenBank accession numbers for DNA sequences and constructs derived from each source are shown, grouped by the indicated genomic locus from which they were amplified. *COI*, cytochrome oxidase subunit 1.

				GENBANK ACCESSION NUMBERS		
**Species**	**Common Name**	**Item and Source**	**Voucher ID**	***COI***	**pCONDEL.329**	**pCONDEL.1189**
Dicentrarchus labrax	European Seabass	Tissue and purified genomic DNA gift from P. Gagnaire and F. Bonhomme (University of Montpellier)	n/a	OP896983 OP896984	OP934176	OP934193 OP934194
Gasterosteus aculeatus	Threespine Stickleback (Little Campbell River)	[lab-raised, this study]	n/a	n/a	n/a	OP934188
Gasterosteus aculeatus	Threespine Stickleback (Matadero Creek)	[lab-raised, this study]	n/a	n/a	n/a	OP934187
Gasterosteus aculeatus	Threespine Stickleback (Salmon River, Rabbit Slough)	Salmon River BAC clone GU130435 [lab-raised, this study]	n/a	n/a	OP934180 OP934181	n/a
Hippocampus erectus	Lined Seahorse	Tissue gift from J. Muniz, A. Seery, & C. Cozzi-Schmarr (Ocean Rider Seahorse Farm, Hawaii)	n/a	OP896990	OP934183 OP934184	OP934185 OP934186
Larimichthys crocea	Large Yellow Croaker	Purchased from 99 Ranch Market in 2019	n/a	OP896988	OP934177 OP934178	OP934189
Mola mola	Ocean Sunfish	Tissue gift from the Ichthyology Collection, University of Kansas, Biodiversity Institute (KUBI Ichthyology)	KU2979	MK144137	OP934174	OP934191
Monopterus albus	Rice Eel	Tissue gift from L. Nico, C. Smith, & S. Collenberg (US Geological Survey)	LGN12-10	OP896985	n/a	OP934192
Oryzias latipes	Japanese Medaka (Hd-rR strain)	Tissue and genomic DNA gift from X. Wang and R. Bhandari (UNC Greensboro)	n/a	OP896987	n/a	OP934173 OP934190
Oryzias latipes	Japanese Medaka OLHNI-2 cell line	RIKEN BioResearch Resource Center (Japan)	RCB2942	OP896986	n/a	n/a
Seriola dumerili	Greater Amberjack	Tissue gift from the Ichthyology Collection, University of Kansas, Biodiversity Institute (KUBI Ichthyology)	KU5141 KU5170	MK144139 MK144135	n/a	OP934195
Syngnathus scovelli	Gulf Pipefish	Genomic DNA gift from S. L. Bassham and W. A. Cresko (University of Oregon)	n/a	OP896989	OP934182	n/a
Takifugu rubripes	Japanese Puffer	Tissue gift from the Ichthyology Collection, University of Kansas, Biodiversity Institute (KUBI Ichthyology)	KU3490	MK144136	OP934200	OP934201
Tetraodon nigroviridis	Green Spotted Puffer	Purchased from AZ Aquatic Gardens [24] in 2019	n/a	OP896980 OP896981 OP896982	OP934175 OP934179 OP934198 OP934199	OP934196 OP934197

Table S3: CRISPR/Cas9 injection mixes.

Injection Group	Component	Final concentration in injection mix (ng/μL)	Associated Oligo ID (see Table S5)
Delete CONDEL region near *Faf1*	Cas9-2NLS	950.00	n/a
	Faf1CONDEL ssDNA	20.00	Faf1CONDEL_ssODN
	FAF1-01 sgRNA	43.75	sgRNA-01
	FAF1-16 sgRNA	150.00	sgRNA-16
	FAF1-17 sgRNA	150.00	sgRNA-17
	Slc24a5 sgRNA	34.15	sgRNA-SLC24A5
Slc24a5 CRISPR knockout control	Cas9-2NLS	950.00	n/a
	Faf1CONDEL ssDNA	20.00	Faf1CONDEL_ssODN
	Slc24a5 sgRNA	377.90	sgRNA-SLC24A5
Faf1 CRISPR knockout	Cas9-2NLS	937.50	n/a
	Slc24a5 sgRNA	40.00	sgRNA-SLC24A5
	FAF1-02 sgRNA	65.00	sgRNA-02
	FAF1-04 sgRNA	65.00	sgRNA-04
	FAF1-32 sgRNA	65.00	sgRNA-32
	FAF1-35 sgRNA	65.00	sgRNA-35

Table S4: Summary statistics for sliding windows used to compute conserved regions.

Genome assembly aligned to oryLat04	Siding window size (10, 25, 50, 100 bp)	Number of tied windows added	Percent identity of least-conserved window included	Total number of windows included after adding ties	Coverage of oryLat04 after including ties
ampCit01	10	14,072,787	90%	32,138,846	7.79%
ampCit01	25	3,865,919	88%	21,312,301	5.82%
ampCit01	50	62,054	86%	17,108,604	5.01%
ampCit01	100	857,808	81%	15,577,367	5.23%
ausLim01	10	3,948,786	90%	23,324,463	5.82%
ausLim01	25	1,558,266	84%	20,978,969	5.29%
ausLim01	50	705,663	80%	19,704,764	5.14%
ausLim01	100	334,819	74%	16,098,009	5.09%
bolPec01	10	5,352,533	80%	25,347,326	6.06%
bolPec01	25	3,346,874	72%	24,963,848	5.56%
bolPec01	50	503,793	70%	20,801,510	5.09%
bolPec01	100	222,797	64%	16,522,092	5.06%
cynSem	10	650,574	90%	19,315,715	5.15%
cynSem	25	2,988,410	80%	22,635,657	5.52%
cynSem	50	1,914,327	76%	20,925,087	5.36%
cynSem	100	490,321	71%	16,080,465	5.12%
dicLab01	10	21,153,704	90%	37,649,954	9.56%
dicLab01	25	7,348,604	88%	23,962,217	6.59%
dicLab01	50	2,764,991	86%	19,201,504	5.62%
dicLab01	100	568,002	83%	14,926,303	5.15%
funHet01	10	4,479,023	90%	24,243,329	5.89%
funHet01	25	3,239,871	84%	22,383,324	5.58%
funHet01	50	2,441,583	80%	20,867,043	5.47%
funHet01	100	177,199	75%	15,450,144	5.04%
gasAcu14	10	8,173,185	90%	26,119,917	6.82%
gasAcu14	25	4,886,414	84%	22,995,029	5.95%
gasAcu14	50	786,697	82%	18,610,779	5.16%
gasAcu14	100	572,246	77%	15,552,985	5.15%
hapBur01	10	14,696,278	90%	32,358,250	8.03%
hapBur01	25	3,407,200	88%	20,825,668	5.74%
hapBur01	50	3,374,405	84%	20,457,531	5.71%
hapBur01	100	677,713	81%	15,417,199	5.18%
hipCom01	10	6,546,452	80%	26,606,146	6.26%
hipCom01	25	468,825	76%	21,971,049	5.08%
hipCom01	50	1,187,502	70%	21,456,403	5.23%
hipCom01	100	135,044	64%	16,262,575	5.03%
hipEre01	10	6,327,045	80%	26,389,929	6.22%
hipEre01	25	247,479	76%	21,777,657	5.04%
hipEre01	50	1,004,461	70%	21,308,876	5.19%
hipEre01	100	70,451	64%	16,231,922	5.02%
kryMar01	10	14,311,684	90%	32,002,824	7.96%
kryMar01	25	3,046,598	88%	20,524,531	5.66%
kryMar01	50	2,856,175	84%	20,041,941	5.60%

Continued on next page

Table S4 – continued from previous page

Genome assembly aligned to oryLat04	Siding window size (10, 25, 50, 100 bp)	Number of tied windows added	Percent identity of least-conserved window included	Total number of windows included after adding ties	Coverage of oryLat04 after including ties
kryMar01	100	187,279	81%	14,940,164	5.05%
labBer01	10	12,756,378	90%	30,217,543	7.82%
labBer01	25	1,121,471	88%	18,518,923	5.25%
labBer01	50	777,279	84%	17,871,905	5.17%
labBer01	100	824,486	79%	15,439,202	5.22%
larCro01	10	19,454,300	90%	37,042,241	9.02%
larCro01	25	5,681,994	88%	23,386,277	6.18%
larCro01	50	1,106,193	86%	18,529,470	5.24%
larCro01	100	452,433	82%	15,557,922	5.12%
latCal01	10	23,416,069	90%	39,658,644	10.04%
latCal01	25	8,861,270	88%	25,247,455	6.91%
latCal01	50	3,960,376	86%	20,172,309	5.89%
latCal01	100	1,297,916	83%	15,485,868	5.36%
mayZeb03	10	13,959,746	90%	32,046,608	7.77%
mayZeb03	25	3,723,013	88%	21,152,282	5.80%
mayZeb03	50	3,730,599	84%	20,708,251	5.78%
mayZeb03	100	685,940	81%	15,301,383	5.18%
miiMii01	10	15,636,572	90%	32,965,956	8.36%
miiMii01	25	3,472,642	88%	20,930,878	5.75%
miiMii01	50	3,289,211	84%	20,497,835	5.69%
miiMii01	100	552,935	81%	15,488,299	5.15%
molMol01	10	10,315,861	90%	27,810,890	7.32%
molMol01	25	6,986,555	84%	24,502,017	6.36%
molMol01	50	2,615,683	82%	19,976,627	5.54%
molMol01	100	27,167	79%	14,803,878	5.01%
monAlb01	10	13,932,349	90%	30,979,175	8.12%
monAlb01	25	1,822,691	88%	18,872,747	5.41%
monAlb01	50	1,778,630	84%	18,703,966	5.38%
monAlb01	100	1,123,912	80%	15,741,178	5.30%
neoBri01	10	12,229,443	90%	30,618,068	7.42%
neoBri01	25	2,537,938	88%	20,232,883	5.54%
neoBri01	50	2,519,111	84%	19,792,998	5.53%
neoBri01	100	907,994	80%	15,700,664	5.24%
notFur02	10	2,975,653	90%	22,970,278	5.59%
notFur02	25	1,826,110	84%	21,390,479	5.33%
notFur02	50	1,420,810	80%	20,300,027	5.27%
notFur02	100	44,489	75%	15,698,318	5.01%
oreNil02	10	15,361,710	90%	33,191,675	8.05%
oreNil02	25	4,676,831	88%	21,924,498	6.00%
oreNil02	50	663,857	86%	17,516,078	5.15%
oreNil02	100	60,859	82%	14,596,238	5.02%
parOli02	10	15,424,400	90%	32,547,196	8.38%
parOli02	25	3,104,008	88%	20,249,671	5.69%
parOli02	50	2,898,380	84%	19,748,597	5.62%

Continued on next page

Table S4 – continued from previous page

Genome assembly aligned to oryLat04	Siding window size (10, 25, 50, 100 bp)	Number of tied windows added	Percent identity of least-conserved window included	Total number of windows included after adding ties	Coverage of oryLat04 after including ties
parOli02	100	257,238	81%	14,742,233	5.07%
poeFor01	10	7,691,039	90%	26,893,407	6.52%
poeFor01	25	6,474,480	84%	24,749,061	6.17%
poeFor01	50	2,337,774	82%	20,025,703	5.47%
poeFor01	100	682,511	77%	15,492,781	5.18%
poeRet02	10	7,659,353	90%	26,759,228	6.52%
poeRet02	25	6,443,158	84%	24,604,838	6.18%
poeRet02	50	2,307,940	82%	19,899,431	5.47%
poeRet02	100	697,501	77%	15,410,228	5.18%
punNye01	10	13,197,344	90%	31,399,819	7.62%
punNye01	25	3,203,697	88%	20,721,252	5.69%
punNye01	50	3,214,249	84%	20,281,700	5.67%
punNye01	100	309,951	81%	14,971,955	5.08%
punPun02	10	9,095,897	90%	26,997,653	7.00%
punPun02	25	5,724,424	84%	23,707,510	6.11%
punPun02	50	1,404,986	82%	19,079,773	5.29%
punPun02	100	848,200	77%	15,742,006	5.22%
serDum01	10	22,996,454	90%	39,270,774	9.95%
serDum01	25	8,685,504	88%	25,087,116	6.86%
serDum01	50	3,888,105	86%	20,149,297	5.87%
serDum01	100	1,385,341	83%	15,602,792	5.38%
serQui01	10	22,366,299	90%	38,715,761	9.82%
serQui01	25	8,213,483	88%	24,701,450	6.77%
serQui01	50	3,505,604	86%	19,827,653	5.78%
serQui01	100	1,084,960	83%	15,360,789	5.30%
synSco01	10	4,981,426	80%	25,381,300	5.95%
synSco01	25	3,300,615	72%	25,061,917	5.55%
synSco01	50	247,749	70%	20,706,083	5.05%
synSco01	100	350,352	63%	16,681,006	5.09%
takFla02	10	1,974,966	90%	20,678,470	5.44%
takFla02	25	5,311,441	80%	24,562,759	5.93%
takFla02	50	1,762,231	78%	20,472,896	5.34%
takFla02	100	448,663	74%	15,921,427	5.11%
takRub01	10	1,955,663	90%	20,692,350	5.44%
takRub01	25	5,262,609	80%	24,562,134	5.92%
takRub01	50	1,681,694	78%	20,452,214	5.32%
takRub01	100	372,479	74%	15,896,061	5.10%
tetNig2	10	11,477,305	80%	31,009,353	7.17%
tetNig2	25	234,709	80%	20,864,475	5.04%
tetNig2	50	1,931,352	74%	21,681,785	5.37%
tetNig2	100	253,266	70%	16,229,452	5.06%
xipCou01	10	119,746	90%	20,560,379	5.02%
xipCou01	25	3,824,517	80%	24,277,168	5.62%
xipCou01	50	402,531	78%	19,863,059	5.08%

Continued on next page

Table S4 – continued from previous page

Genome assembly aligned to oryLat04	Siding window size (10, 25, 50, 100 bp)	Number of tied windows added	Percent identity of least-conserved window included	Total number of windows included after adding ties	Coverage of oryLat04 after including ties
xipCou01	100	7,460	69%	15,919,720	5.00%
xipHel01	10	9,957,730	80%	31,189,076	6.43%
xipHel01	25	1,889,845	80%	22,959,004	5.30%
xipHel01	50	758,896	76%	20,661,567	5.14%
xipHel01	100	99,664	64%	16,301,809	5.02%
xipMac02	10	9,027,389	90%	27,849,819	6.81%
xipMac02	25	244,169	88%	18,078,229	5.05%
xipMac02	50	139,890	84%	17,469,325	5.03%
xipMac02	100	802,355	78%	15,399,347	5.21%

Table S5: Oligonucleotides used in this study.

Oligo ID	Oligo Sequence	Description	Species
Faf1CONDEL_ssODN	5' G*G*CTCGCTTAAACCAGCAGTCAAAGCCCAAC-TACTGCTCTATTTCTGTGGGAAATGTT*C*G 3'	ssODN for pCONDEL.1189 CRISPR; *=phosphorothioate bond	Threespine Stickleback
sgRNAscaffoldOligo	5' GATCCGCACCGACTCGGTGCCACTTTTTCAAGTTGATAACG-GACTAGCCTTATTTTAACTTGCTATTTCTAGCTCTAAAAC 3'	for 2-oligo PCR to generate sgRNA template	n/a
129,589.F_y	CCTTTCACTGTGYAATCAGCTC	amplification, sequencing primer	[multiple species]
129,611.R_r	GAGCTGATTRCACAGTGAAAGG	amplification, sequencing primer	[multiple species]
dicLab-1189seq-R2	CTGATGATCCCCAGCTCCTG	amplification, sequencing primer	European Seabass
dicLab-1189seq-R3	AGGCATCCGTGTTTGAAAGC	amplification, sequencing primer	European Seabass
dicLab-CONDEL1189-F1	TGCCCTGTGCTTTACCCTTT	amplification, sequencing primer	European Seabass
dicLab-CONDEL1189-R1	CTGCAAGGTGTCTCCAGAGG	amplification, sequencing primer	European Seabass
dicLab-PelA-F0	GGTGCCAGGAAGAGTAGCTG	amplification, sequencing primer	European Seabass
dicLab-PelA-F1	CCCTGTGTCATGTCTAAGGCA	amplification, sequencing primer	European Seabass
dicLab-PelA-F3	GTGTCCTATCCATCAGCTGT	amplification, sequencing primer	European Seabass
dicLab-PelA-F4	TACTGTCTGTGATCGCTCTC	amplification, sequencing primer	European Seabass
dicLab-PelA-F5	CCTGTGTGCTGTCAGTGGTA	amplification, sequencing primer	European Seabass
dicLab-PelA-pGL4.23-fwd	AGCTCGCTAGCCTCGAGGATTTGAGAGAGGCCTGGAGAAG	primer for Gibson Assembly into pGL4.23 vector	European Seabass
dicLab-PelA-pGL4.23-rev	TATATACCCTCTAGTGTCTAGTCCCCTCCTCTATTTTTAA	primer for Gibson Assembly into pGL4.23 vector	European Seabass
dicLab-PelA-R1	GGGGGATCCTGCACTTTGAA	amplification, sequencing primer	European Seabass
dicLab-PelA-R2	ACCCAAGTGTGTGTGTGTGT	amplification, sequencing primer	European Seabass
dicLab-PelA-R3	TGCCTTAGACATGACACAGGG	amplification, sequencing primer	European Seabass
dicLab-PelA-R3.5	CAGCTACTCTTCCTGGCACC	amplification, sequencing primer	European Seabass
dicLab-PelA-R4	ACAATGCACCGTACAGCTCA	amplification, sequencing primer	European Seabass
dicLab-PelA-R5	TCAAACGCATCAGATGGCCT	amplification, sequencing primer	European Seabass
dicLab-PelAseq-F2	ACCTCAGGTCACAGGTTTTCT	amplification, sequencing primer	European Seabass
dicLab-PelAseq-R2	AGTGGCAACGTACAGCAAGA	amplification, sequencing primer	European Seabass
FAF1-ex5-R1	ACTGTGCTGCCTTCCTCCA	amplification, sequencing primer	Threespine Stickleback
FAF1-in1-F1	CCTGTTGTGGGTTGGTACCA	amplification, sequencing primer	Threespine Stickleback
FAF1-in2-F1	CGTGCTTGGCTTTCAGCATT	amplification, sequencing primer	Threespine Stickleback
FAF1-in2-R2	TAGGAACTTGGCGGACACAC	amplification, sequencing primer	Threespine Stickleback
FAF1-in3-F2	TGTCTCCTTCAGTGTTCGGT	amplification, sequencing primer	Threespine Stickleback
FAF1-in3-R1	CAACCACTGTAATCATGTCGCT	amplification, sequencing primer	Threespine Stickleback
fr-CONDEL1189-F1	AAGGTCTCCCCAGGTGGATT	amplification, sequencing primer	Japanese Puffer
fr-CONDEL1189-R1	GGATTCCTGTGCTTCAGCCT	amplification, sequencing primer	Japanese Puffer

Continued on next page

Table S5 – continued from previous page

Oligo ID	Oligo Sequence	Description	Species
fr-CONDEL1189seq-F2	TAAGCATTGACCTCTGCTGG	amplification, sequencing primer	Japanese Puffer
fr-CONDEL1189seq-R2	AACGAGCCACTCTACCATGC	amplification, sequencing primer	Japanese Puffer
fr3-PelA-F2	TCACTTCGCAGCTCGTATCC	amplification, sequencing primer	Japanese Puffer
fr3-PelA-fwdAnchor	TAATGAAAAGGCCTTAAATCCCTCT	amplification, sequencing primer	Japanese Puffer
fr3-PelA-revAnchor	TGTCACCCCAGAGTTATAAAAACTG	amplification, sequencing primer	Japanese Puffer
fr3-PelAseq-F1	GGGTTCGGCCTGGTGATTAA	amplification, sequencing primer	Japanese Puffer
fr3-PelAseq-R1	TGACCCCAAGCTCCATTACG	amplification, sequencing primer	Japanese Puffer
gasAcu-CONDEL1189-F1	GCGTTGGAATAAGCAGCGAG	amplification, sequencing primer	Threespine Stickleback
gasAcu-CONDEL1189-R1	GCAAGGTGTTAGCAGAGGGT	amplification, sequencing primer	Threespine Stickleback
gasAcu-CONDEL1189-R2	ACACAGACTGGTTTGTGGTCCA	amplification, sequencing primer	Threespine Stickleback
gasAcu-CONDEL1189seq-F2	CACAGCAATGAACGGCACAA	amplification, sequencing primer	Threespine Stickleback
gasAcu-CONDEL1189seq-R2	TGTTGGAGGAGCCGATGTTC	amplification, sequencing primer	Threespine Stickleback
gasAcu-crispr1189-F1	CCTACTCCTTGTCTCGGCCAAA	amplification, sequencing primer	Threespine Stickleback
gasAcu-crispr1189-R2	ACACAGACTGGTTTGTGGTCCA	amplification, sequencing primer	Threespine Stickleback
HC M13 forward	GTAAAACGACGGCCAGTGAA	vector backbone sequencing primer	n/a [vector backbone]
HC M13 reverse	GGAAACAGCTATGACCATGA	vector backbone sequencing primer	n/a [vector backbone]
HC Sp6 promoter primer	CTATTTAGGTGACACTATAG	vector backbone sequencing primer	n/a [vector backbone]
HC T7 promoter primer	TAATACGACTCACTATAGGG	vector backbone sequencing primer	n/a [vector backbone]
HC_pT2HE_rev	CATATAGACAAAACATGCTCGTTC	vector backbone sequencing primer	n/a [vector backbone]
hipCom-CONDEL1189-F1	TTTGAAGAGTGGAGGTGCCC	amplification, sequencing primer	Lined Seahorse
hipCom-CONDEL1189-R1	TGAAGGTGTCCCCAGAAGGT	amplification, sequencing primer	Lined Seahorse
hipCom-PelA-F1	ATCGAGTCAACCAAACCGCT	amplification, sequencing primer	Lined Seahorse
hipCom-PelA-R1	GGCCTGACACTGTCCAGAAG	amplification, sequencing primer	Lined Seahorse
hipEre-PelA-F1	CCCCCTAGTGACCAAGCAAG	amplification, sequencing primer	Lined Seahorse
hipEre-PelAseq-F2	ATCGGCAAACGTTTTGCTTGCTC	amplification, sequencing primer	Lined Seahorse
hipEre-PelAseq-F3	GACACTCGCCTCTTCAATAACC	amplification, sequencing primer	Lined Seahorse
hipEre-PelAseq-R1	CAGTGAGCCAAGTCCATCAA	amplification, sequencing primer	Lined Seahorse
larCro-CONDEL1189-F1	AGGGATGAGGATGAGCAGGA	amplification, sequencing primer	Yellow Croaker
larCro-CONDEL1189-F2	GAGCAGGAGAAATGTTAATGATCAAT	amplification, sequencing primer	Yellow Croaker
larCro-CONDEL1189-F3	CCAGCTCCAGTGCAGAAAGA	amplification, sequencing primer	Yellow Croaker
larCro-CONDEL1189-F4	GCCACTGGATCACTGTCACA	amplification, sequencing primer	Yellow Croaker
larCro-CONDEL1189-F5	TGGAGAGGAGCAATGCACAG	amplification, sequencing primer	Yellow Croaker
larCro-CONDEL1189-F6	GTCATTCACAGTCCGAGGCA	amplification, sequencing primer	Yellow Croaker
larCro-CONDEL1189-F7	AAAAGGTTCAGTGCCAGGCA	amplification, sequencing primer	Yellow Croaker
larCro-CONDEL1189-F8	GCAACAGATTGGACCCCTGT	amplification, sequencing primer	Yellow Croaker
larCro-CONDEL1189-F9	TGCCATGAAAATGGAGCCCT	amplification, sequencing primer	Yellow Croaker
larCro-CONDEL1189-R1	GCCTTTGTCAGAGAAGCGCT	amplification, sequencing primer	Yellow Croaker
larCro-CONDEL1189-R2	CAACACAGCAGGTATGGCAC	amplification, sequencing primer	Yellow Croaker
larCro-CONDEL1189-R3	ACAGGGGTCCAATCTGTTGC	amplification, sequencing primer	Yellow Croaker
larCro-CONDEL1189-R4	GCATCAGCCTTTTCACAGCC	amplification, sequencing primer	Yellow Croaker
larCro-CONDEL1189-R5	CCCATCTGCCAATGGTCACT	amplification, sequencing primer	Yellow Croaker
larCro-CONDEL1189-R6	TGTGACAGTGATCCAGTGGC	amplification, sequencing primer	Yellow Croaker
larCro-PelA-3prime	CCCTACTCCTCTATTTTTAA	amplification, sequencing primer	Yellow Croaker
larCro-PelA-5prime	TTGAGAGAGGCCTGGAGAGG	amplification, sequencing primer	Yellow Croaker
larCro-PelA-F3.1	GATTTACATTTGCCAGCTGG	amplification, sequencing primer	Yellow Croaker
larCro-PelA-F3.2	TAAAAGACTACACGGTCTCT	amplification, sequencing primer	Yellow Croaker
larCro-PelA-F4.1	CTCTCTCCTTCCAAGTATTCCAAG	amplification, sequencing primer	Yellow Croaker
larCro-PelA-innerFwdAnchor	ATTTGACCTTTGTCCCCTCCC	amplification, sequencing primer	Yellow Croaker
larCro-PelA-innerRevAnchor	AAAAGTGATGTAAACATTGAGAGAGGC	amplification, sequencing primer	Yellow Croaker
larCro-PelA-outerFwdAnchor	TAATGATAAGGCCTTAAATCCCTCTG	amplification, sequencing primer	Yellow Croaker
larCro-PelA-outerRevAnchor	GTGTGCAGTACTTGGGCTCT	amplification, sequencing primer	Yellow Croaker
larCro-PelA-pGL4.23-fwd	AGCTCGCTAGCCTCGAGGATTTGAGAGAGGCCTGGAGAGG	primer for Gibson Assembly into pGL4.23 vector	Yellow Croaker
larCro-PelA-pGL4.23-rev	TATATACCCTCTAGTGTCTAGTCCCCTCCCCCCTACTCCT	primer for Gibson Assembly into pGL4.23 vector	Yellow Croaker
larCro-PelA-pT2HE-GA-fwd	GGCCCGATATCACCGGGGGATTGAGAGAGGCCTGGAGAGG	primer for Gibson Assembly into pT2HE vector	Yellow Croaker

Continued on next page

Table S5 – continued from previous page

Oligo ID	Oligo Sequence	Description	Species
larCro-PelA-pT2HE-GA-rev	CAAGCGACACCCCTGAAGGAGTCCCCTCCCCCCTACTCCT	primer for Gibson Assembly into pT2HE vector	Yellow Croaker
larCro-PelA-R1	TGACAAGCCCACACACTGTT	amplification, sequencing primer	Yellow Croaker
larCro-PelA-R1.5	GTCTGGGCTCTGCTCGTTTG	amplification, sequencing primer	Yellow Croaker
larCro-PelA-R1.75	CACAATTAATCAGTGGGCCAAA	amplification, sequencing primer	Yellow Croaker
larCro-PelA-R2	GGCCAAATGCACAATGGGTT	amplification, sequencing primer	Yellow Croaker
larCro-PelA-R3	CGCAAAGCCCTCCTACTGAA	amplification, sequencing primer	Yellow Croaker
larCro-PelA-R4	TGAGGCAAGGGGGTTGTAAC	amplification, sequencing primer	Yellow Croaker
larCro-PelA-R4.1	CTTGGAATACTTGGAAGGAGAGAG	amplification, sequencing primer	Yellow Croaker
larCro-PelA-R5	ATTCTCTTTGACCCTGCCGG	amplification, sequencing primer	Yellow Croaker
larCro-PelA-R6	TCATTTAGAAATCTTGGATATCTAA	amplification, sequencing primer	Yellow Croaker
larCro-PelAseq-F0	CACAGCCACGTTCCAAACAC	amplification, sequencing primer	Yellow Croaker
larCro-PelAseq-F0.5	TTTGGCCCACTGATTAATTGTG	amplification, sequencing primer	Yellow Croaker
larCro-PelAseq-F1	AACCCATTGTGCATTTGGCC	amplification, sequencing primer	Yellow Croaker
larCro-PelAseq-F2	TTCAGTAGGAGGGCTTTGCG	amplification, sequencing primer	Yellow Croaker
larCro-PelAseq-F2.5	GAGGTGTCATTTTCTGTGAGGAG	amplification, sequencing primer	Yellow Croaker
larCro-PelAseq-F3	AACAGTGTGTGGGCTTGTCA	amplification, sequencing primer	Yellow Croaker
larCro-PelAseq-F4	GTTACAACCCCCTTGCCTCA	amplification, sequencing primer	Yellow Croaker
larCro-PelAseq-F5	CCGGCAGGGTCAAAGAGAAT	amplification, sequencing primer	Yellow Croaker
larCro-PelAseq-F6	GTGAAAGGATCCCTCGGTGT	amplification, sequencing primer	Yellow Croaker
larCro-PelAseq-F7	GCGGTGAGGTGAATACGGAT	amplification, sequencing primer	Yellow Croaker
larCro-PelAseq-F8	CCACCTTCCCAGATCTCAGC	amplification, sequencing primer	Yellow Croaker
larCro0-PelA-R3.1	CTCCTCACAGAAAATGACACCTC	amplification, sequencing primer	Yellow Croaker
luc2-forMinP	GTCCACCTCGATATGTGCGT	amplification, sequencing primer	Yellow Croaker
luc2rev	CCCTTCTTAATGTTTTTGGC	vector backbone sequencing primer	n/a [vector backbone]
minP-rev	ATTGCCAAGCTGGAAGTCGA	vector backbone sequencing primer	n/a [vector backbone]
molMol-CONDEL1189-F1	GGTACAGATGGCGTTCGGAA	amplification, sequencing primer	Ocean Sunfish
molMol-CONDEL1189-R1	AGGGGTAAGGATCAGCAGGA	amplification, sequencing primer	Ocean Sunfish
molMol-PelA-F1	GCTGCAGCGTTCTTTGATGT	amplification, sequencing primer	Ocean Sunfish
molMol-PelA-F6	TTTTCCACACACGCCTAGCT	amplification, sequencing primer	Ocean Sunfish
molMol-PelA-F7	TCCAATCAAATTCCATTCAGCCG	amplification, sequencing primer	Ocean Sunfish
molMol-PelA-fwdAnchor	TGTCATCCCAGAGTTATAAAAAGTGA	amplification, sequencing primer	Ocean Sunfish
molMol-PelA-pGL4.23-fwd	AGCTCGCTAGCCTCGAGGATCTGAGCGGGGCCTGGATAGG	primer for Gibson Assembly into pGL4.23 vector	Ocean Sunfish
molMol-PelA-pGL4.23-rev	TATATACCCTCTAGTGTCTAGTCCCGTCTATTTTTAAAAC	primer for Gibson Assembly into pGL4.23 vector	Ocean Sunfish
molMol-PelA-R1	CCTGTGTCATTGGGTCCACA	amplification, sequencing primer	Ocean Sunfish
molMol-PelA-R5	AAGTCAGTGATCCAGCTGGC	amplification, sequencing primer	Ocean Sunfish
molMol-PelA-R6	GGGGTGGCATGTTTGTTGAG	amplification, sequencing primer	Ocean Sunfish
molMol-PelA-revAnchor	TAATGGTAAGGCCTTAAATCCCCC	amplification, sequencing primer	Ocean Sunfish
molMol-PelAseq-F2	CTCAACAAACATGCCACCCC	amplification, sequencing primer	Ocean Sunfish
molMol-PelAseq-F3	GTGCTTCTCCTGCAAAAGGC	amplification, sequencing primer	Ocean Sunfish
molMol-PelAseq-F4	GCCAGCTGGATCACTGACTT	amplification, sequencing primer	Ocean Sunfish
molMol-PelAseq-F5	TCTGGGCTTTTTGGGTGTGT	amplification, sequencing primer	Ocean Sunfish
molMol-PelAseq-R2	TCCACTCATGTGATGCCCTG	amplification, sequencing primer	Ocean Sunfish
molMol-PelAseq-R3	CACTGATTGTAACAGTTGTCGC	amplification, sequencing primer	Ocean Sunfish
molMol-PelAseq-R4	GCAGCACTTTGGAGGTCCTC	amplification, sequencing primer	Ocean Sunfish
monAlb-1189-F2	CTGCTCATATCCAGGCCCAG	amplification, sequencing primer	Rice Eel
monAlb-1189-R2	CCGAGTCAAAACGGTCTCCA	amplification, sequencing primer	Rice Eel
monAlb-1189seq-F1	GAGACGAGGGTTCCATGCAA	amplification, sequencing primer	Rice Eel
monAlb-1189seq-R1	ACCCTCTTCTCTGCCTTCCT	amplification, sequencing primer	Rice Eel
monAlb-CONDEL1189-F1	GCAGCAAGAGGAGAGATGCA	amplification, sequencing primer	Rice Eel
monAlb-CONDEL1189-R1	GGGCAAGGATCAGCAAGAGA	amplification, sequencing primer	Rice Eel
oryLat-CONDEL1189-F1	ATACTGACAGTGATGCGGCA	PCR amplification, sequencing primer	Japanese Medaka
oryLat-CONDEL1189-F2	ACCGCAGTGAAATCAGCCTT	PCR amplification, sequencing primer	Japanese Medaka
oryLat-CONDEL1189-F3	CAATGCAACGGAGACACACC	PCR amplification, sequencing primer	Japanese Medaka
oryLat-CONDEL1189-F4	TTTCCTCCCTCTCGCCTTTCTG	PCR amplification, sequencing primer	Japanese Medaka

Continued on next page

Table S5 – continued from previous page

Oligo ID	Oligo Sequence	Description	Species
oryLat-CONDEL1189-R1	GGATCAGGGTGGCTTCAAGTGA	PCR amplification, sequencing primer	Japanese Medaka
oryLat-CONDEL1189-R2	CCGTGCCTCTCGTGGTAAAT	PCR amplification, sequencing primer	Japanese Medaka
oryLat-CONDEL1189-R3	TTTAGTGCTCTCCGTGCCTC	PCR amplification, sequencing primer	Japanese Medaka
oryLat-CONDEL1189-R4	TCATGGATCAGGGTGGCTTCAA	PCR amplification, sequencing primer	Japanese Medaka
oryLat-CONDEL1189-preNheIfwd3	AGATAGGCCCCTTACGTACGCTCAATGCAACGGAGACACACC	primer for Gibson Assembly into pT2HE vector	Japanese Medaka
oryLat-CONDEL1189-postNheIrev1	CCGGTGATATCGGGCCCGCTGGATCAGGGTGGCTTCAAGTGA	primer for Gibson Assembly into pT2HE vector	Japanese Medaka
oryLat-DEL963-F1	CACATTTCTGCAAGGTGCCC	PCR amplification, sequencing primer	Japanese Medaka
oryLat-DEL963-F3	TGGACACTTGTACAGGCCACAT	PCR amplification, sequencing primer	Japanese Medaka
oryLat-DEL963-F4	TGACAGATTCCCATTTGCATGTGT	PCR amplification, sequencing primer	Japanese Medaka
oryLat-DEL963-F5	AGTCGTCATGAGGATTCCCTTT	PCR amplification, sequencing primer	Japanese Medaka
oryLat-DEL963-F6	AGGGGACTGAAAGAGGAAAGTTGT	PCR amplification, sequencing primer	Japanese Medaka
oryLat-DEL963-F7	CCGTGTTCATTTCAGGGGCAAT	PCR amplification, sequencing primer	Japanese Medaka
oryLat-DEL963-F8	TCTGGACCGTTCGAATCAAGCT	PCR amplification, sequencing primer	Japanese Medaka
oryLat-DEL963-F9	TGTGGAGGTTTGACTGACTGCT	PCR amplification, sequencing primer	Japanese Medaka
oryLat-DEL963-R10	ATTGCCCCTGAAATGAACACGG	PCR amplification, sequencing primer	Japanese Medaka
oryLat-DEL963-R11	ACAACTTTCCTCTTTCAGTCCCCT	PCR amplification, sequencing primer	Japanese Medaka
oryLat-DEL963-R3	CTTTAGCCTCTGTCAGAACGCG	PCR amplification, sequencing primer	Japanese Medaka
oryLat-DEL963-R4	CCCTCCATGTGGCCTGTACAAG	PCR amplification, sequencing primer	Japanese Medaka
oryLat-DEL963-R5	TGAGCTGGAAATGGAGACTCGT	PCR amplification, sequencing primer	Japanese Medaka
oryLat-DEL963-R8	AGCTTGATTCGAACGGTCCAGA	PCR amplification, sequencing primer	Japanese Medaka
oryLat-DEL963-R9	GGGTCCTTCCAACTGTTTGCAC	PCR amplification, sequencing primer	Japanese Medaka
oryLat-PelA-F1	GGGGAATCCTGCACTTTGAA	amplification, sequencing primer	Japanese Medaka
oryLat-PelA-F5	CTCTAGTCTGATATTGAAGCGTATT	amplification, sequencing primer	Japanese Medaka
oryLat-PelA-F6	TTAAGCTCCTGATCGCCGCT	amplification, sequencing primer	Japanese Medaka
oryLat-PelA-fwdAnchor	TGTCACTCCAGAGTTCTAAAGAGT	amplification, sequencing primer	Japanese Medaka
oryLat-PelA-pGL4.23-fwd	AGCTCGCTAGCCTCGAGGATCTGAAAGTGAGGCCAGGAGA	primer for Gibson Assembly into pGL4.23 vector	Japanese Medaka
oryLat-PelA-pGL4.23-rev	TATATACCCTCTAGTGTCTAGTCCTCCTTTTTTCTCTATT	primer for Gibson Assembly into pGL4.23 vector	Japanese Medaka
oryLat-PelA-R1	CGTTCTTCCTTCATCGCTGC	amplification, sequencing primer	Japanese Medaka
oryLat-PelA-R4	GGTCTGCAGGTGACACGTAA	amplification, sequencing primer	Japanese Medaka
oryLat-PelA-revAnchor	GATAAAGCCCTAAATCCTTCTGTAAA	amplification, sequencing primer	Japanese Medaka
oryLat-PelAseq-F2	TGCGTCTGTTCGTGCAAAAG	amplification, sequencing primer	Japanese Medaka
oryLat-PelAseq-F3	CATGCTTACAGCCCCTCACA	amplification, sequencing primer	Japanese Medaka
oryLat-PelAseq-F4	TTACGTGTCACCTGCAGACC	amplification, sequencing primer	Japanese Medaka
oryLat-PelAseq-R2	ACCCTCGGTTTAAGCATTAAGGT	amplification, sequencing primer	Japanese Medaka
oryLat-PelAseq-R3	AGCCCATCGGAACCTTTCAG	amplification, sequencing primer	Japanese Medaka
PelA 129,078.F	CATCACCGAGCCGCTTTGAT	amplification, sequencing primer	Threespine Stickleback
PelA 129,096.F	ATGTGGGCCTAATATGGCTTG	amplification, sequencing primer	Threespine Stickleback
PelA 129,165.R	TCTGGACAGCGTCAGGCAAC	amplification, sequencing primer	Threespine Stickleback
PelA 129,231.F	GTCTGCAGCTGTCATCCTG	amplification, sequencing primer	Threespine Stickleback
PelA 129,237.F	CTGTCATCCTGGAGTTATAAAAC	amplification, sequencing primer	Threespine Stickleback
PelA 129,262.F	CATCCTGGAGTTATAAAACGTG	amplification, sequencing primer	Threespine Stickleback
PelA 129,282.F	GATGTAAACATTGAGAGGGTC	amplification, sequencing primer	Threespine Stickleback
PelA 129,334.F	CTGTCATGGAGCACCACCTC	amplification, sequencing primer	Threespine Stickleback
PelA 129,345.F	CACCACCTCGTTCCTGAAGG	amplification, sequencing primer	Threespine Stickleback
PelA 129,345.R	CCTTCAGGAACGAGGTGGTG	amplification, sequencing primer	Threespine Stickleback
PelA 129,410.F	CCATTTACTAAAAATGCTCAACTC	amplification, sequencing primer	Threespine Stickleback
PelA 129,433.R	GAGTTGAGCATTTTTAGTAAATGG	amplification, sequencing primer	Threespine Stickleback
PelA 129,451.F	CTCGATTTCCATCACGTTGTT	amplification, sequencing primer	Threespine Stickleback
PelA 129,490.F	GCACATGAAGGATCATTAACAG	amplification, sequencing primer	Threespine Stickleback
PelA 129,504.R	GTGCACCTGAATTACTGTTAATG	amplification, sequencing primer	Threespine Stickleback
PelA 129,508.F	CAGTAATTCAGGTGCACAAAC	amplification, sequencing primer	Threespine Stickleback
PelA 129,560.F	CAGCCTGATGTGCAGCACAC	amplification, sequencing primer	Threespine Stickleback
PelA 129,656.R	AGCTTATCTCGGCTGTTTATGT	amplification, sequencing primer	Threespine Stickleback
PelA 129,689.F	GTCGAAGCAAAGAGGCGAG	amplification, sequencing primer	Threespine Stickleback

Continued on next page

Table S5 – continued from previous page

Oligo ID	Oligo Sequence	Description	Species
PelA 129,750.R	GATATCTGAATGTTTATGAATAACA	amplification, sequencing primer	Threespine Stickleback
PelA 129,754.F	CCAACAAGAAACTGTTCAAATG	amplification, sequencing primer	Threespine Stickleback
PelA 129,918.F	TCAGGGCCGGACCATCTAA	amplification, sequencing primer	Threespine Stickleback
PelA 129,968.R	GCAGAGTTCTAAAGTGGTCG	amplification, sequencing primer	Threespine Stickleback
PelA 129,976.R	GACCTTGTGCAGAGTTCTAAAG	amplification, sequencing primer	Threespine Stickleback
PelA 129,985.R	ATGCATTAGGACCTTGTGCAG	amplification, sequencing primer	Threespine Stickleback
PelA 129,987.F	CTGTTTGACCTCGCCGGAG	amplification, sequencing primer	Threespine Stickleback
PelA 129,993.R	CAAACAGAATGCATTAGGACC	amplification, sequencing primer	Threespine Stickleback
PelA 129,999.R	CGAGGTCAAACAGAATGCATTAG	amplification, sequencing primer	Threespine Stickleback
PelA 129.937.F	CTCTTCCACTGATTGTTATG	amplification, sequencing primer	Threespine Stickleback
PelA 130,000.F	CCGGAGTAAATCAAATACTGG	amplification, sequencing primer	Threespine Stickleback
PelA 130,020.R	CCAGTATTTGATTTACTCCGG	amplification, sequencing primer	Threespine Stickleback
PelA 130,164.F	CACGCTAGACACAAGGAAGG	amplification, sequencing primer	Threespine Stickleback
PelA 130,250.R	GTGACCACAACAATCCGTGG	amplification, sequencing primer	Threespine Stickleback
PelA 130,269.F	CCACGGATTGTTGTGGTCAC	amplification, sequencing primer	Threespine Stickleback
PelA 130,322.F	AGCTAGCCGCTAACAGGTAG	amplification, sequencing primer	Threespine Stickleback
PelA 130,377.F	AGTAGCGTTCAACTCTTTCTAG	amplification, sequencing primer	Threespine Stickleback
PelA 130,398.R	CTAGAAAGAGTTGAACGCTACT	amplification, sequencing primer	Threespine Stickleback
PelA 130,485.R	GGTGGTTATTTAAATAGAGACAATA	amplification, sequencing primer	Threespine Stickleback
PelA 130,520.R	GACATGCTGGTCTATCAGAC	amplification, sequencing primer	Threespine Stickleback
PelA 130,547.F	GTGTTCTTCATAATACAGAATCAGCATC	amplification, sequencing primer	Threespine Stickleback
PelA 130,577.R	GAAGATGCTGATTCTGTATTATG	amplification, sequencing primer	Threespine Stickleback
PelA 130,625.R	CACGGAGGACGTCTTTCAGG	amplification, sequencing primer	Threespine Stickleback
PelA 130,634.R	GACAACTCGCACGGAGGAC	amplification, sequencing primer	Threespine Stickleback
PelA 130,643.F	GTACTGCGATAGATCTGAGG	amplification, sequencing primer	Threespine Stickleback
PelA 130,662.R	CCTCAGATCTATCGCAGTAC	amplification, sequencing primer	Threespine Stickleback
PelA 130,665.R	GGACCTCAGATCTATCGCAG	amplification, sequencing primer	Threespine Stickleback
PelA 130,757.R	CTCCTGCTGGAGGACCTTAG	amplification, sequencing primer	Threespine Stickleback
PelA 130,778.R	GTTTGTCAGCAGAGAGGTTTC	amplification, sequencing primer	Threespine Stickleback
PelA 130,877.F	CACGCCCCGTCCTCCCGA	amplification, sequencing primer	Threespine Stickleback
PelA 130,920.R	TTTTTATTTTGATATGTCTCCTGC	amplification, sequencing primer	Threespine Stickleback
PelA 130,943.R	AAATCCCTCTGTAGATTTGACC	amplification, sequencing primer	Threespine Stickleback
PelA 130,957.R	CGATAAGGCCTTAAATCCCTC	amplification, sequencing primer	Threespine Stickleback
PelA 131,454.R	GAAATGAGAACATTTCACATCTAC	amplification, sequencing primer	Threespine Stickleback
PelA 131,480.F	GTCTGAAATATGTTATAGGTGC	amplification, sequencing primer	Threespine Stickleback
PelA 131,644.R	TCATGTTGTAGAATGAATTGAGC	amplification, sequencing primer	Threespine Stickleback
PelA 131,675.F	CCTGCAGTAAATAACTAGGAG	amplification, sequencing primer	Threespine Stickleback
PelA 131,713.R	ATAGTTTAGTATAGTATACTCCTAG	amplification, sequencing primer	Threespine Stickleback
PelA 131,758.R	TCTCTCAGCGGAGAAATCCG	amplification, sequencing primer	Threespine Stickleback
PelA 132,268.R	AGCTTCGTCACGCCACCTG	amplification, sequencing primer	Threespine Stickleback
PelA_insertion_fwd1	GCGTGTGATTGCCAGAGACATT	amplification, sequencing primer	Threespine Stickleback
PelA_insertion_fwd2	GTGTGGCTGGTCTCGTATCATA	amplification, sequencing primer	Threespine Stickleback
PelA_insertion_rev1	GAATGTTAATAATTTAAGGCTGT	amplification, sequencing primer	Threespine Stickleback
PelA_insertion_rev1fwd	ACAGCCTTAAATTATTAACATTC	amplification, sequencing primer	Threespine Stickleback
PelA_insertion_rev2	ATAATTTAAGGCTGTCGGGGGT	amplification, sequencing primer	Threespine Stickleback
PelA_insertion_rev3	AATGTCTCTGGCAATCACACGC	amplification, sequencing primer	Threespine Stickleback
PelA_insertion_rev4	CTCATGGGGGAATGTTAATA	amplification, sequencing primer	Threespine Stickleback
percomorph-COI-fwd	ttctccaccaaccacaargayatygg	amplification, sequencing primer	[multiple species]
percomorph-COI-rev	cacctcagggtgtccgaaraaycaraa	amplification, sequencing primer	[multiple species]
RVprimer3	TAGCAAAATAGGCTGTCCC	vector backbone sequencing primer	n/a [vector backbone]
SALR-PelA-pGL4.23-fwd	AGCTCGCTAGCCTCGAGGATTTGAGAGGGTCTGGAGGAGC	primer for Gibson Assembly into pGL4.23 vector	Threespine Stickleback
SALR-PelA-pGL4.23-rev	TATATACCCTCTAGTGTCTATTTATTTTGATATGTCTCCT	primer for Gibson Assembly into pGL4.23 vector	Threespine Stickleback
SALR-PelA-pT2HE-GA-fwd	GGCCCGATATCACCGGGGGATTGAGAGGGTCTGGAGGAGC	primer for Gibson Assembly into pT2HE vector	Threespine Stickleback
SALR-PelA-pT2HE-GA-rev	CAAGCGACACCCCTGAAGGATTTATTTTGATATGTCTCCT	primer for Gibson Assembly into pT2HE vector	Threespine Stickleback

Continued on next page

Table S5 – continued from previous page

Oligo ID	Oligo Sequence	Description	Species
seqLuc2fwd	TAGACACTAGAGGGTATATAATGGA	vector backbone sequencing primer	n/a [vector backbone]
seqLuc2rev	CTGCATTCTAGTTGTGGTTTGTC	vector backbone sequencing primer	n/a [vector backbone]
seqLuc2revMid	CCAGTGTCTTACCGGTGTCC	vector backbone sequencing primer	n/a [vector backbone]
seqSV40polyA_fwd	GCAAGATCGCCGTGTAATAA	vector backbone sequencing primer	n/a [vector backbone]
serDum-CONDEL1189-F1	CTGCAAGGTGTCCTCAGAGG	amplification, sequencing primer	Greater Amberjack
serDum-CONDEL1189-F2	CCCACCTGCCTCAAAACTGA	amplification, sequencing primer	Greater Amberjack
serDum-CONDEL1189-F3	GCACGCACAGGTTGAATTGT	amplification, sequencing primer	Greater Amberjack
serDum-CONDEL1189-F4	TTCCTCCTGAACTGGGGACT	amplification, sequencing primer	Greater Amberjack
serDum-CONDEL1189-R1	TTTGAAGAGTGGAGGTGCCC	amplification, sequencing primer	Greater Amberjack
serDum-CONDEL1189-R2	CCCTGTGCTTTAGCCTCTGT	amplification, sequencing primer	Greater Amberjack
serDum-CONDEL1189-R3	AGCTCCCGTGTTGATCACTG	amplification, sequencing primer	Greater Amberjack
serDum-PelA-F0	AATCTTTACCAGGGGCCTGC	amplification, sequencing primer	Greater Amberjack
serDum-PelA-F0.5	TGCCAAGTTCCCCACATCAA	amplification, sequencing primer	Greater Amberjack
serDum-PelA-F1	TCCTTTTTCTGGGCTCACGG	amplification, sequencing primer	Greater Amberjack
serDum-PelA-pGL4.23-fwd	AGCTCGCTAGCCTCGAGGATTTGGGAGAGGCCTGGAGAAA	primer for Gibson Assembly into pGL4.23 vector	Greater Amberjack
serDum-PelA-pGL4.23-rev	TATATACCCTCTAGTGTCTATTTGTCCCCTCCTCTATTTT	primer for Gibson Assembly into pGL4.23 vector	Greater Amberjack
serDum-PelA-R1	GGGCGATCCTGCTCTTTGAA	amplification, sequencing primer	Greater Amberjack
serDum-PelA-R3	CCGTGAGCCCAGAAAAAGGA	amplification, sequencing primer	Greater Amberjack
serDum-PelAseq-F2	CTGTAGCAGGGTTAGTGCCA	amplification, sequencing primer	Greater Amberjack
serDum-PelAseq-R2	TGGCCAGTAATGGTTGCTGT	amplification, sequencing primer	Greater Amberjack
sgRNA-01	AATTAATACGACTCACTATAGGAATCAATAGACTGGGCCGGTTTTAGAGCTAGAAATAGC	for 2-oligo PCR to generate sgRNA template	Threespine Stickleback
sgRNA-02	AATTAATACGACTCACTATAGGTGCAGCAACTTCCCCAGTGGTTTTAGAGCTAGAAATAG	for 2-oligo PCR to generate sgRNA template	Threespine Stickleback
sgRNA-04	AATTAATACGACTCACTATAGGCCGGATGCTCAACTTCCGGGGTTTTAGAGCTAGAAATA	for 2-oligo PCR to generate sgRNA template	Threespine Stickleback
sgRNA-16	AATTAATACGACTCACTATAGGAAATAGAGCAGTAGAGACAGTTTTAGAGCTAGAAATAG	for 2-oligo PCR to generate sgRNA template	Threespine Stickleback
sgRNA-17	AATTAATACGACTCACTATAGGCAGTCAAAGCCCAAGATCAGTTTTAGAGCTAGAAATAG	for 2-oligo PCR to generate sgRNA template	Threespine Stickleback
sgRNA-32	AATTAATACGACTCACTATAGGTGGTAAGTTCTTTATCTGTGTTTTAGAGCTAGAAATAG	for 2-oligo PCR to generate sgRNA template	Threespine Stickleback
sgRNA-35	AATTAATACGACTCACTATAGGTCATTACAGGCAGCCATCAAGTTTTAGAGCTAGAAATA	for 2-oligo PCR to generate sgRNA template	Threespine Stickleback
sgRNA-SLC24A5	AATTAATACGACTCACTATAGAAGCCGTCGGGGAACTCGGGTTTTAGAGCTAGAAATAGC	for 2-oligo PCR to generate sgRNA template	Threespine Stickleback
synSco-CONDEL1189-F1	AGCACACACCAGGGATGTAC	amplification, sequencing primer	Gulf Pipefish
synSco-CONDEL1189-R1	AGCGCAGACTTTGAAGAGCT	amplification, sequencing primer	Gulf Pipefish
synSco-PelA-F1	TGTTGTTACTGCCCCCTAGC	amplification, sequencing primer	Gulf Pipefish
synSco-PelA-fwdAnchor	TGAAGTTAAGGCCCTTAAATCCC	amplification, sequencing primer	Gulf Pipefish
synSco-PelA-pGL4.23-fwd	AGCTCGCTAGCCTCGAGGATCAAAAGAAAGAGTCTGGATA	primer for Gibson Assembly into pGL4.23 vector	Gulf Pipefish
synSco-PelA-pGL4.23-rev	TATATACCCTCTAGTGTCTAGAACCTTCCCCTCATCCCCT	primer for Gibson Assembly into pGL4.23 vector	Gulf Pipefish
synSco-PelA-R1	GCACTTCATCGCAGTTCTCG	amplification, sequencing primer	Gulf Pipefish
synSco-PelA-R1	GCACTTCATCGCAGTTCTCG	amplification, sequencing primer	Gulf Pipefish
synSco-PelA-revAnchor	TGTCATTCCGGAGTTATAAACGT	amplification, sequencing primer	Gulf Pipefish
synSco-PelA-seq-R1	AAGGCGGGATATGCGTCAAA	amplification, sequencing primer	Gulf Pipefish
tetNig-CONDEL1189-F1	TCTCCCCAGGTGCATTAGGA	amplification, sequencing primer	Green Spotted Puffer
tetNig-CONDEL1189-R1	GGATTCCTGTGCTTCAGCCT	amplification, sequencing primer	Green Spotted Puffer
tetNig-PelA-3prime	GTCCCGTCTATTTTTAAACC	amplification, sequencing primer	Green Spotted Puffer
tetNig-PelA-5prime	CTGAGAAAAGTCTGGAGAAG	amplification, sequencing primer	Green Spotted Puffer
tetNig-PelA-fwdAnchor	TGTGACCCCAGAGTTATAAAAACTG	amplification, sequencing primer	Green Spotted Puffer
tetNig-PelA-pGL4.23-fwd	AGCTCGCTAGCCTCGAGGATCTGAGAAAAGTCTGGAGAAG	primer for Gibson Assembly into pGL4.23 vector	Green Spotted Puffer
tetNig-PelA-pGL4.23-rev	TATATACCCTCTAGTGTCTAGTCCCGTCTATTTTTAAACC	primer for Gibson Assembly into pGL4.23 vector	Green Spotted Puffer
tetNig-PelA-pT2HE-GA-fwd	GGCCCGATATCACCGGGGGACTGAGAAAAGTCTGGAGAAG	primer for Gibson Assembly into pT2HE vector	Green Spotted Puffer
tetNig-PelA-pT2HE-GA-rev	CAAGCGACACCCCTGAAGGAGTCCCGTCTATTTTTAAACC	primer for Gibson Assembly into pT2HE vector	Green Spotted Puffer
tetNig-PelA-R1	GAGTTCACCGTACAGCCTTC	amplification, sequencing primer	Green Spotted Puffer
tetNig-PelA-revAnchor	TGGAAAGGCCTTAAATCCCTCT	amplification, sequencing primer	Green Spotted Puffer
tetNig-PelAseq-F1	TTTGCTGGCTACGTCTCAGG	amplification, sequencing primer	Green Spotted Puffer

Table S6: Sources of images and illustrations in Figure 1.

Species	Type of Depiction	Artist/Creator	Source of Digital Copy or Tissue	Copyright Holder	Usage Rights
European Seabass	illustration	G. Cuvier M. Valenciennes	[25]	Public Domain	[not under license]
Green Spotted Puffer	photo of stained caudal skeleton (image inset paired with Japanese Puffer)	H. I. Chen D. M. Kingsley	purchased specimen [24]	[authors of this study]	n/a
Gulf Pipefish	illustration	J. Tomelleri	[26]	J. Tomelleri	usage rights purchased
Japanese Medaka	illustration	D. Jordan, C. Metz	[27]	Public Domain	[not under license]
Japanese Puffer	illustration	B. Yau	[28]	B. Yau	[29]
Lined Seahorse	radiograph	S. Raredon	[30]	Public Domain	[not under license]
Ocean Sunfish	illustration	H. Smith	[31]	Public Domain	[not under license]
Ocean Sunfish	illustration (of skeleton)	J. Steenstrup C. Lütken	[15]	Public Domain	[not under license]
Rice Eel	illustration	F. Day	[32]	Public Domain	[not under license]
Rice Eel	photo of stained caudal skeleton	H. I. Chen D. M. Kingsley	Leo Nico Specimen LGN 12-10	[authors of this study]	n/a
Threespine Stickleback	illustration	E. Edmonson	[33]	NY Department of Environmental Conservation	CC BY-NC-ND 2.0
Tongue Sole	illustration	D. Jordan, C. Metz	[27]	Public Domain	[not under license]
Tongue Sole	caudal radiograph	D. Loffler	[34]	Public Domain	[not under license]
Yellow Croaker	illustration	B. Yau	[35]	B. Yau	[29]

References

[1] R. Fricke, D. Golani, and B. Appelbaum-Golani. *Cynoglossus crepida*, a new species of tonguesole from the Gulf of Aqaba, Red Sea (Teleostei: Cynoglossidae). *Journal of the Ocean Science Foundation*, 25:77–87, 2017. doi: 10.5281/zenodo.321395. URL https://zenodo.org/record/321395.

[2] A. G. K. Menon. *A systematic monograph of the tongue soles of the genus* Cynoglossus *Hamilton-Buchanan (Pisces, Cynoglossidae)*. Smithsonian Institution Press, Washington D.C., 1977. doi: 10.5479/si.00810282.238. URL https://repository.si.edu/handle/10088/5354.

[3] T. A. Munroe. *The living marine resources of the Western Central Pacific*. FAO species identification guide for fishery purposes. Food and Agriculture Organization of the United Nations, Rome, 2001. ISBN 92-5-104589-5. URL https://www.fao.org/3/y0870e/y0870e52.pdf. Chapter: 'Cynoglossidae - tonguesoles'.

[4] R. Fricke. *Cynoglossus westraliensis*, a new species of tonguesole from Western Australia (Teleostei: Cynoglossidae). *FishTaxa*, 4(2):31–40, 2019. URL https://www.fishtaxa.com/index.php/ft/article/viewFile/4-2-3/135.

[5] T. Naito and H. Endo. A new tongue sole of the genus *Cynoglossus* from the East China Sea and Yellow Sea (Pleuronectiformes: Cynoglossidae). *Ichthyological Research*, 66(3):400–410, 2019. doi: 10.1007/s10228-019-00685-x. URL https://doi.org/10.1007/s10228-019-00685-x.

[6] C. Daoulas, A. N. Economou, and I. Bantavas. Osteological abnormalities in laboratory reared sea-bass (*Dicentrarchus labrax*) fingerlings. *Aquaculture*, 97(2):169–180, 1991. doi: 10.1016/0044-8486(91)90263-7. URL https://www.sciencedirect.com/science/article/pii/0044848691902637.

[7] G. Marino, C. Boglione, B. Bertolini, A. Rossi, F. Ferreri, and S. Cataudella. Observations on development and anomalies in the appendicular skeleton of sea bass, *Dicentrarchus labrax* L. 1758, larvae and juveniles. *Aquaculture Research*, 24(3):445–456, 1993. doi: 10.1111/j.1365-2109.1993.tb00568.x. URL https://onlinelibrary.wiley.com/doi/abs/10.1111/j.1365-2109.1993.tb00568.x.

[8] P. Konstantinidis and G. D. Johnson. A comparative ontogenetic study of the tetraodontiform caudal complex. *Acta Zoologica*, 93(1):98–114, 2012. ISSN 1463-6395. doi: 10.1111/j.1463-6395.2010.00490.x. URL https://onlinelibrary.wiley.com/doi/abs/10.1111/j.1463-6395.2010.00490.x.

[9] C. C. Lindsey. Experimental study of meristic variation in a population of threespine sticklebacks, *Gasterosteus aculeatus*. *Canadian Journal of Zoology*, 40(2):271–312, 1962. doi: 10.1139/z62-028.

[10] T. H. Huxley. Observations on the development of some parts of the skeleton of fishes. *Transactions of The Microscopical Society & Journal*, 7(1):33–46, 1859. ISSN 1365-2818. doi: 10.1111/j.1365-2818.1859.tb04575.x. URL http://onlinelibrary.wiley.com/doi/abs/10.1111/j.1365-2818.1859.tb04575.x.

[11] J. S. Nelson, T. Grande, and M. V. H. Wilson. *Fishes of the world*. John Wiley & Sons, Hoboken, New Jersey, 5th edition, 2016. ISBN 978-1-118-34233-6.

[12] T. Franz-Odendaal and D. Adriaens. Comparative developmental osteology of the seahorse skeleton reveals heterochrony amongst *Hippocampus* sp. and progressive caudal fin loss. *EvoDevo*, 5(45):1–11, 2014. doi: 10.1186/2041-9139-5-45.

[13] Q.-R. Wang, Y.-Y. Ni, L.-M. Lin, and Z.-Y. Wang. Development of the vertebral column and the pectoral and caudal fins in larvae of yellow croaker *Larimichthys crocea* (Richardson). *Acta Hydrobiologica Sinica*, 34(3):467–472, 2010. doi: 10.3724/SP.J.1035.2010.00467.

[14] J. C. Tyler. *Osteology, phylogeny, and higher classification of the fishes of the order Plectognathi (Tetraodontiformes).* U.S. Department of Commerce, National Oceanic and Atmospheric Administration, National Marine Fisheries Service, Seattle, WA, 1980. doi: 10.5962/bhl.title.63022. URL https://www.biodiversitylibrary.org/item/128327.

[15] J. J. S. Steenstrup and C. F. Lütken. *Spolia atlantica. Bidrag til kundskab om klump- eller maanefiskene (Molidae).* Bianco Lunos Kgl. Hof-Bogrrykkeri (F. Dreyer), Kjøbenhavn, 1898. URL https://www.biodiversitylibrary.org/bibliography/13309.

[16] U. S. Allen. *Monopterus albus* (Asian swamp eel). Datasheet, CAB International, 2019. URL https://www.cabi.org/isc/datasheet/74114.

[17] Monarch. *Monopterus albus* (Zuiew, 1793) radiograph collection, 2022. URL https://monarch.calacademy.org/taxa/index.php?tid=411998&taxauthid=1&clid=0.

[18] M. Y. Ali and C. C. Lindsey. Heritable and temperature-induced meristic variation in the medaka, *Oryzias latipes*. *Canadian Journal of Zoology*, 52(8):959–976, 1974. doi: 10.1139/z74-128. URL http://www.nrcresearchpress.com/doi/10.1139/z74-128.

[19] E. F. Balart. Development of median and paired fin skeleton of *Paralichthys olivaceus* (Pleuronectiformes: Paralichthyidae). *Japanese Journal of Ichthyology*, 31(4):398–410, 1985. doi: 10.11369/jji1950.31.398. See Table 1.

[20] N. Okada, M. Tanaka, and M. Tagawa. Bone development during metamorphosis of the Japanese flounder (*Paralichthys olivaceus*): differential responses to thyroid hormone. In *The Big Fish Bang*, 26th Annual Larval Fish Conference, pages 177–187, Norway, 2003. Institute of Marine Research. ISBN 82-7461-059-8. URL https://citeseerx.ist.psu.edu/pdf/de88bf68fd2309c63f840d4bf13b8cada3028f6b. See Figure 4C-D.

[21] J. M. D. d. Astarloa, T. A. Munroe, P. Béarez, M. Gonzalez-Castro, and D. L. Castellini. External morphology, postcranial and appendicular osteology of three southwestern Atlantic flatfishes (*Paralichthys*, Paralichthyidae), and comparisons with other congeneric species. *Neotropical Ichthyology*, 16(2):e170164, 2018. doi: 10.1590/1982-0224-20170164. URL http://www.scielo.br/j/ni/a/gZnjGzc7kHC5xsKmvbCCxpt/?lang=en.

[22] A. Laggis, D. G. Sfakianakis, P. Divanach, and M. Kentouri. Ontogeny of the body skeleton in *Seriola dumerili* (Risso, 1810). *Italian Journal of Zoology*, 77(3):303–315, 2010. doi: 10.1080/11250000903170870. URL http://www.tandfonline.com/doi/abs/10.1080/11250000903170870.

[23] Z. Chen, Y. Zhang, Z. Han, N. Song, and T. Gao. Morphological characters and DNA barcoding of *Syngnathus schlegeli* in the coastal waters of China. *Journal of Oceanology and Limnology*, 36(2):537–547, 2018. doi: 10.1007/s00343-017-6206-2. URL http://link.springer.com/10.1007/s00343-017-6206-2.

[24] Green Spotted Puffer Fish, 2019. URL https://azgardens.com/product/green-spotted-puffer-fish/.

[25] G. Cuvier and M. Valenciennes. *Histoire naturelle des poissons.* Chez F. G. Levrault, Paris, 1828. URL https://www.biodiversitylibrary.org/page/7111258. Plate 11.

[26] J. Tomelleri. American Fishes, 2019. URL https://www.americanfishes.com/en/content/13-about-contact.

[27] D. S. Jordan, C. W. Metz, and Carnegie Museum. *A catalog of the fishes known from the waters of Korea*. Board of Trustees of the Carnegie Institute, Pittsburgh, 1913. URL https://archive.org/details/catalogoffishesk61jord/page/24/mode/1up.

[28] B. Yau. *Takifugu rubripes*, year of creation unknown. URL https://www.efishalbum.com/details.asp?pid=798.

[29] B. Yau. Fish album terms & conditions, 2022. URL https://www.efishalbum.com/memtermsofuse.asp.

[30] S. J. Raredon. USNM 155442 *Hippocampus erectus* radiograph, year of creation unknown. URL http://n2t.net/ark:/65665/m3cdd9a677-65bc-428e-acee-f55d93aa76bf.

[31] H. M. Smith. *Fishes of North Carolina*. E.M. Uzzell & Co., North Carolina, 1907. URL https://archive.org/details/fishesofnorthcar02smit/page/352/mode/2up.

[32] F. Day. *The fishes of India; being a natural history of the fishes known to inhabit the seas and fresh waters of India, Burma, and Ceylon*, volume 2. B. Quaritch, London, 1878. URL https://www.biodiversitylibrary.org/page/5616157#page/355/mode/1up. Plate CLXIX.

[33] E. Edmonson. Threespine stickleback, 2010. URL https://www.flickr.com/photos/nysdec/29865050421/.

[34] D. Loffler. *Areliscus rhomaleus* RAD107247-001, 1994. URL http://n2t.net/ark:/65665/m3aa836717-9caa-4dba-9318-d64275603924.

[35] B. Yau. *Pseudosciaena crocea*, year of creation unknown. URL https://www.efishalbum.com/details.asp?pid=801.
